# The Mediating Role of Internalized Stigma and Shame on the Relationship between COVID-19 Related Discrimination and Mental Health Outcomes among Back-to-School Students in Wuhan

**DOI:** 10.3390/ijerph17249237

**Published:** 2020-12-10

**Authors:** Hao Li, Ling Zheng, Hong Le, Lijun Zhuo, Qian Wu, Guoqing Ma, Hongbing Tao

**Affiliations:** School of Medicine and Health Management, Tongji Medical College, Huazhong University of Science and Technology, Wuhan 430030, China; leohao@hust.edu.cn (H.L.); zhengling@hust.edu.cn (L.Z.); lehong@hust.edu.cn (H.L.); lijunzhuo@hust.edu.cn (L.Z.); hustayla7@hust.edu.cn (Q.W.); mgq@hust.edu.cn (G.M.)

**Keywords:** discrimination, internalized stigma, shame, mental health, college students, COVID-19

## Abstract

Outbreaks of an epidemic, such as coronavirus disease 2019 (COVID-19), always brings about far-ranging discrimination and stigmatization to the epicenter. This was a cross-sectional survey conducted to assess experienced discrimination, internalized stigma, shame, and mental health (anxiety, depression, distress, insomnia) among college students who merely had a perceived linkage with COVID-19, and explore the linkage between discrimination and negative mental health outcomes through the mediating effects of shame and internalized stigma. A total of 995 participants (53% female) were involved in this study, in which 40.9% of college students were reported to be discriminated against because of their experience in Wuhan. The experience of COVID-19-related discrimination is indirectly associated with anxiety, depression, and insomnia, in which shame and internalized stigma play a complete mediating effect. Meanwhile, it is both directly and indirectly associated with distress through shame and internalized stigma. The findings of this study suggest that COVID-19-related discrimination is associated with shame and internalized stigma, which in turn predict psychological symptoms over time.

## 1. Introduction

At the end of December 2019, a novel pneumonia caused by coronavirus disease 2019 (COVID-19) was first identified in Wuhan; it soon escalated into a global health emergency [1,2]. The world faced an unprecedented public health crisis. Apart from physical health, mental health was equally crucial and worthy of attention. Epidemic outbreaks have been historically accompanied by stigma, discrimination, and xenophobia [3], leading to psychological harm to individuals in the epicenter [4]. Previous studies have elucidated a few interactions of these psychological issues. Moreover, the linkage between COVID-19 and racism brought about anti-Chinese activities, and these acts of discrimination occurred in social, political, and historical contexts [5]. Social media bias against Asian Americans increased, with escalated radical and abusive expressions, which incurred discrimination and adverse mental health consequences [6]. Such COVID-19-related discrimination was quite evident and omnipresent among individuals, especially those who manifested a potential linkage with Wuhan during the outbreak of COVID-19, since Wuhan was the first epicenter of this global health crisis. Consequently, those discriminated individuals became more vigilant concerning stigma-related threats, as well as more vulnerable to stigmatization [7]. However, little information is available that reveals how invisible harm caused by discrimination exerts its toll on mental health during the COVID-19 pandemic. Therefore, underlying psychological mechanisms of COVID-19-related discrimination associated with mental health needs to be further explored.

Stigma is a personal attribute that is socially devalued [8] and exists with the co-occurrence of labeling, stereotyping, social exclusion, discrimination [9]. Importantly, stigma is context-specific and not deemed to reside in the person but in a social context [10,11]. According to the theory of the stigma framework, a stigma is generally categorized into discrimination and internalized stigma [12]. Therefore, in the context of the COVID-19 pandemic, internalized stigma refers to the awareness of devaluation or a stereotype of oneself because of a perceived linkage with COVID-19 [12,13]. In an overview of outbreaks of epidemics during past decades, epidemic-related stigma unremittingly occurred in particular groups, which could be a barrier to the containment of the epidemic, leading to detrimental consequences for psychological wellbeing [14,15]. Wuhan, the initial epicenter of COVID-19, was put under an international spotlight, leading to the stigmatized label “Wuhan virus” [16]. In addition to the mandatory quarantine measures, media-driven misinformation and overloaded news reports ignited fear and uncertainty, provoking stigma and discrimination. According to the COVID-19 Health Stigma and Discrimination Framework (HSDF) [17], the experience of discrimination, along with shame, were drivers of stigmatization, and the internalized stigma can be regarded as a manifestation to exert a negative impact on mental health outcomes. Besides, internalized stigma could manifest as self-hatred, self-isolation, shame, and fear of further stigmatization [18,19,20]. The empirical study, demonstrating perceived epidemic-related discrimination, was far-ranging and increased with time. As a result, it might profoundly contribute to long-term adverse mental health outcomes [21,22]. Moreover, individuals with travel experience in the epicenter were affected by internalized stigma [23], and that could be psychologically damaging for individuals affected by COVID-19, since high levels of internalized stigma might be associated with lower levels of self-esteem and increased depression [10,24]. In addition, previous studies have shown the association between multifaceted discrimination and retention cares in HIV-positive young adolescents; this process was mediated by internalized stigma [25]. Although the repercussion of internalized stigma has been widely revealed, the psychological process was still unclear in the context of COVID-19.

Shame is a negative self-conscious emotion [26], triggered when individuals experience failure in regards to personal or social standards. Ashamed individuals might blame themselves rather than others, believing that failures reflect inadequacy in themselves. Shame often originates from incorrect perception or belief in particular events, and it may be an underlying mechanism in which stigma is enacted [27]. Concerning infectious disease-perceived discrimination, individuals may exhibit feelings of shame and stigmatization synchronously. Moreover, shame has been shown to mediate the negative mental and physical health consequences of stigmatization, both shame and stigma are directly related to mental health problems [25,28,29]. However, they probably have different manifestations. In particular, shame is a part of the experience of which individuals seek health-care services, while stigma may be a barrier in decisions to seek care [30]. Previous research indicated that individuals with sexually transmitted diseases (STD), HIV/AIDs, hepatitis C, and mental illnesses were more likely to suffer poor mental and physical health if they felt ashamed of their stigma. Often in cases where infectious disease-related shame was a strong predictor of stigma [31], previous studies demonstrated that shame mediated the relationship between stigma and depression [32]. Unfortunately, up until now, researches focused on shame in COVID-19, and the psychological effects of this process are scarce. We could not make a firm conclusion of the psychological impact so far.

To date, regarding the increasing concern about mental health during the post-COVID-19 pandemic, the present study sought to access the knowledge gap of the mental health status and address the issue related to discrimination, shame, and stigma connected with mental health.

## 2. Methods

### 2.1. Setting and Participants

This was a cross-sectional study using a web-based survey to assess the experiences of COVID-19-related discrimination, shame, internalized stigma with mental health outcomes among college students. Participants who were not exposed to the risk of COVID-19, but merely had a perceived linkage with the first epicenter of COVID-19, were invited to participate in this survey, from 31 August to 14 September, 2020. The snowball sampling method, which focused on recruiting college students in Wuhan, was used to obtain samples. The sample size should be 5–10 times the number of scale entries. Furthermore, it was expected that at least 696 participants would be required, considering a sample dispersion rate of 20%. Participants were encouraged to participate in online surveys or complete offline questionnaires. Eligibility criteria of this study included: (i) participants who were studying in Wuhan before the COVID-19 outbreak (as well as currently in Wuhan); (ii) participants aged 18 years or older; (iii) participants who volunteered to take part in this study.

### 2.2. Procedure

This was an anonymous survey, and data confidentiality were ensured. We developed the online questionnaires on the official website of “Questionnaire Star”, which is recognized as a professional online questionnaire survey platform. To practice social distancing and avoid large-scale gatherings, we printed the informed consent form with the attached QR code. Once the QR code (via a mobile phone) was scanned, participants could directly enter the survey page and then fill out the questionnaire. The informed consent forms from all participants were obtained before they completed the online questionnaires, and the web-based survey prompted participants to complete skipped items. Therefore, no missing item-level data were present during the process of data collection. After removing participants with dozens of consecutive identical and illogical item responses, our effective sample included 995 participants.

### 2.3. Measures

Internalized stigmatization: it was measured by a 12-item scale adapted from Visser and colleagues [33]. It included two dimensions, identified as “Blame and Judgement” and “Interpersonal Distancing”. Stigma is time- and context-specific [10]; therefore, the items were tailored to reflect the context of COVID-19-related internalized stigma. The Chinese version of this scale was translated in a previous study by Li et al. [23], showing adequate reliability and validity for reflecting participant levels of internalized stigma. Items were rated on a 7-point Likert-type scale, and scales with higher scores indicated a higher level of internalized stigma. The Cronbach’s α for the scale in this study was 0.806.

Shame: it was measured by a 6-item scale, adapted from Fortenberry and colleagues [34]. A bilingual researcher was recruited to perform the translation from English to Chinese, and then we conducted a backward translation for confirmation. Higher total scores reflect the higher level of shame. The Cronbach’s α for the scale in this study was 0.899.

Anxiety: it was assessed by the Chinese version [35] of Generalized Anxiety Disorder Scale-7 (GAD-7), which comprised 7 self-reported symptoms rating over the past two weeks. The GAD-7 used a Likert-type frequency scale from “0 = Not at all” to “3 = Nearly every day.” The Cronbach’s α coefficient of the Chinese version of GAD-7 is 0.898, and the test-retest reliability coefficient was 0.856, proving it had good reliability and validity in the application of evaluating anxiety. In addition, summed scores were reliable and valid [36]. We tailored this instruction to query COVID-19-related anxiety, specifying “Over the last 2 weeks, how often have you been bothered by the following problems because of the outbreak of COVID-19?”. The total score ranged from 0 to 21, with higher scores indicating a greater degree of anxiety. The Cronbach’s α for the scale in this study was 0.928.

Depression: it was measured by the Chinese version of Patient Health Questionnaire-9 (PHQ-9), which was a self-rating measurement assessing depressive symptoms over the last two weeks. The PHQ-9 used a 4-point Likert scale from 0 (never) to 3 (nearly every day), with higher scores indicating a greater severe symptom of depression. This scale has been widely used among the Chinese, indicating excellent psychometric properties [37]. The Cronbach’s α for the scale in this study was 0.917.

Insomnia: the severity of insomnia was measured by the Chinese version of the Insomnia Severity Index scale (ISI-7) [38]. It consisted of seven items, rated on a five-point Likert scale, including evaluating sleep onset and maintenance, early morning awakening, sleep dissatisfaction, interference of sleep difficulties with daytime functioning, noticeability of sleep problems by others, and distress caused by disturbed sleep. The total score ranged from 0 to 28, with higher scores indicating a greater severity of insomnia. The Cronbach’s α for the scale in this study was 0.903.

Distress: COVID-19-related distress was measured by the Chinese version of the Impact of Event Scale (IES-7) with a seven-item subscale [39]. The Chinese version of IES-7 used a 6-point Likert scale from 0 “never” to 5 “a high degree” with higher IES total scores, indicating participants with greater self-reported severe symptoms. The items were designedly tailored to reflect the context of COVID-19 related distress. The Cronbach’s α for the scale in this study was 0.805.

The experience of COVID-19-related discrimination: we used a “yes”/“no” binary item to query COVID-19-related discrimination [40,41] by asking, “Have you ever been discriminated against because of your experience of studying and living in Wuhan, Hubei province?”. Moreover, we subsequently used a series of multiple choice questions to query the main sources and forms of discrimination by asking, “If you are discriminated against or treated differently, what are the main sources from and in which form?”.

Covariates: the following covariates were included in this study: gender, age, and years expected to graduate.

### 2.4. Common Method Bias

Common method bias analysis was conducted before data analysis. The Eigenvalues of 11 factors were greater than 1. The first factor explains 29.34% of the accumulative variation, which indicated no serious common method bias deviation.

### 2.5. Data Analysis

The data were calculated and analyzed by SPSS version 24.0 (IBM SPSS, IBM Corp., Armonk, NY, USA). Apart from descriptive statistics and frequency analysis of demographic characteristics and the COVID-19-related information, we conducted a t-test or (F-test) and correlation test to examine relationships of potential covariates with our predictors, mediators, and outcomes. These analyses guided our covariate selections. Prior to testing for mediation, we examined the possibility that shame and internalized stigma may moderate the relationship between the experience of discrimination and detrimental consequences for psychological wellbeing. Consequently, preliminary analyses did not confirm the moderating effects. After adjusting for age, gender and, years expected to graduate, the mediation models were conducted by using the bootstrapping procedure by Preacher and Hayes, and the corresponding SPSS macro Model 6 within SPSS 24.0 to test whether COVID-19-related shame and internalized stigma mediated the relationship between discrimination and mental health problems. We utilized non-standardized coefficients and bootstrapping with 5000 samples to place 95% bias-corrected confidence intervals around estimates.

## 3. Results

### 3.1. Characteristics of Participants

The overview of sociodemographic characteristics and the experience of discrimination relative information are exhibited in Table 1. It is shown that 40.90% of college students were reported to be discriminated against because of their experiences in Wuhan. The main discrimination sources were from the community (76.66%), friends and relatives (54.30%), and social media (32.92%). The main forms of discrimination are social avoidance (68.06%), personal information leakage (54.05%), and abusive expressions (45.95%).

### 3.2. Correlation between Variables

Means, standard deviations, and bivariate correlations of variables in this study are shown in Table 2. The experience of discrimination, shame, internalized stigma, and negative mental health outcomes (anxiety, distress, depression, and insomnia) are positively correlated. In particular, the experience of discrimination is positively correlated with shame (r = 0.116, *p* < 0.001), internalized stigma (r = 0.390, *p* < 0.001), anxiety (r = 0.084, *p* < 0.01), distress (r = 0.175, *p* < 0.001), depression (r = 0.099, *p* < 0.01) and insomnia (r = 0.101, *p* < 0.01). Moreover, similar results are observed in shame and internalized stigma.

### 3.3. Mediating Effect Analysis

Table 3 illustrates the linear regression analysis results, and the control variables are sex, age, and year expected to graduate. Regression analysis reveals that the experience of discrimination could positively predict higher shame (β = 0.7683, *p* < 0.05), internalized stigma (β = 7.811, *p* < 0.001), and distress (β = 0.7698, *p* < 0.05). Moreover, as shown in Table 3, shame can positively predict internalized stigma (β = 1.1975, *p* < 0.001), anxiety (β = 0.2636, *p* < 0.001), distress (β = 0.2536, *p* < 0.001), depression (β = 0.3848, *p* < 0.001), and insomnia (β = 0.3038, *p* < 0.001). Internalized stigma can positively predict anxiety (β = 0.0552, *p* < 0.001), distress (β = 0.0787, *p* < 0.001), depression (β = 0.0569, *p* < 0.001) and insomnia (β = 0.0670, *p* < 0.001).

**Figure 1:**
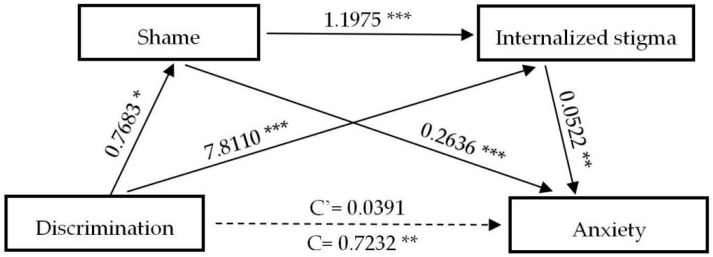
A serial mediation analysis of shame and internalized stigma on discrimination to anxiety. * *p* < 0.05, ** *p* < 0.01, *** *p* < 0.001. C: direct effect without mediators; C’: direct effect with the mediators of shame and internalized stigma.

**Figure 2:**
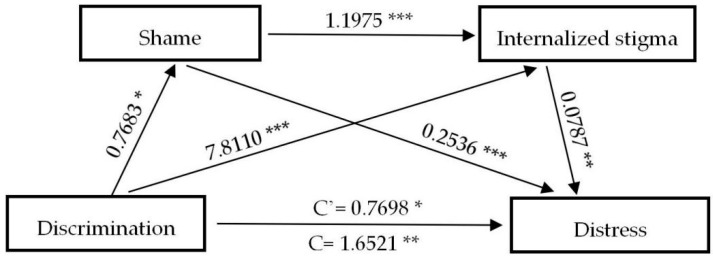
A serial mediation analysis of shame and internalized stigma on discrimination to distress. * *p* < 0.05, ** *p* < 0.01, *** *p* < 0.001.

**Figure 3:**
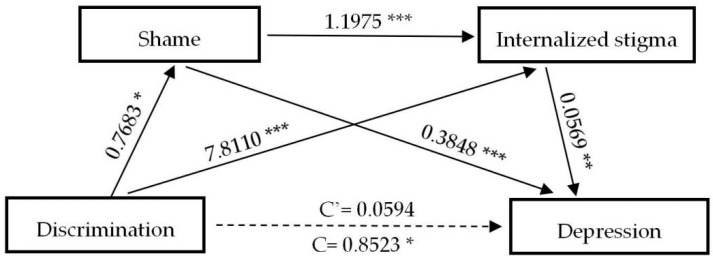
A serial mediation analysis of shame and internalized stigma on discrimination to depression. * *p* < 0.05, ** *p* < 0.01, *** *p* < 0.001.

**Figure 4:**
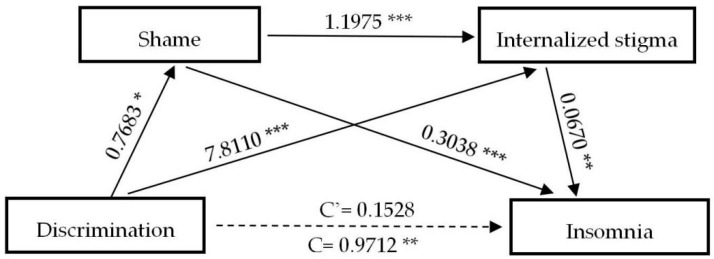
A serial mediation analysis of shame and internalized stigma on discrimination to insomnia. * *p* < 0.05, ** *p* < 0.01, *** *p* < 0.001.

Table 4 exhibits the results of direct and indirect paths and effects of the experience of discrimination on the negative psychological outcomes, respectively. Notably, the bootstrap method indicates that except distress (β = 0.7689, (CI: 0.1501, 1.3896)), the experience of discrimination does not exert direct effects (C’) on psychological outcomes assessed, while none of the direct effects of anxiety (β = 0.0391, (CI: −0.5020, 0.5802)), depression (β = 0.0594, (CI: −0.5759, 0.6948)) and insomnia (β = 0.1528, (CI: −0.5372, 0.8428)) are statistically significant. However, the total indirect effect of discrimination on anxiety (β = 0.6841, (CI: 0.3889, 0.9918)), distress (β = 0.8822, (CI: 0.5511, 1.2642)), depression (β = 0.7929, (CI: 0.4261, 1.1704)) and insomnia (β = 0.8184, (CI: 0.4580, 1.2168)) are significantly observed. Specifically, shame and internalized stigma have played complete mediating roles in predicting anxiety, depression, and insomnia; three indirect paths between the experience of discrimination and negative mental health outcomes (NMHO) are significant: (a) D→S→NMHO, (b) D→IS→NMHO, (c) D→S→IS→NMHO, and total indirect effects overwhelmingly accounted for 94.59%, 93.03%, and 84.27% of the total effect, respectively. After controlling for shame and internalized stigma, the experience of discrimination can significantly predict distress, indicating that shame and internalized stigma are partial mediators between the experience of discrimination and distress. The total indirect effect accounts for 53.40% of the total effect. To better understand the associations between the experience of discrimination and negative mental health outcomes, Figure 1, Figure 2, Figure 3 and Figure 4 are plotted to depict the indirect and their chain mediation paths.

The coefficients shown above are non-standardized. Shame and internalized stigma in sequence mediated the relationship between discrimination and anxiety, distress, depression, and insomnia.

## 4. Discussion

Epidemic outbreaks have been historically accompanied by stigma, discrimination, and xenophobia, leading to psychological harm to individuals in the epicenter [4]. Such stigma and discrimination were quite evident among college students in Wuhan since China was the first country afflicted by the COVID-19 pandemic, and Wuhan was the first epicenter of the unprecedented global health crisis [1,2]. However, it was quite challenging to uncover the underlying mechanisms of the toll on mental health outcomes and reveal certain invisible harm caused by discrimination and stigma. The focus of this cross-sectional study is twofold: (1) to investigate the experience of COVID-19-related discrimination among returning college students in Wuhan and explore the linkage between discrimination and negative mental health outcomes; and (2) to assess the mediating role of shame and internalized stigma between the experiences of COVID-19-related discrimination and negative mental health outcomes.

To our knowledge, this is the first quantitative study to examine COVID-19-related discrimination experienced by Chinese back-to-school (returning) college students. Our study demonstrates nearly 41% of them were reported to have encountered different kinds of discrimination because of their experience in Wuhan, which is in line with a study conducted among Hong Kong residents during the outbreak of Severe Acute Respiratory Syndrome (SARS) [42]. Additionally, 76.7%, 54.3%, and 32.9% of them were discriminated against in various ways (e.g., social avoidance, personal information leakage, abusive expressions, deliberately complicated examination procedures, body conflict, etc.) by their local communities, friends, relatives, and social media, respectively. This might be associated with uncertainty from the public towards infectious diseases and Chinese nationwide over-mobilization [16], which, turned out to be useful in the containment of COVID-19. Likewise, during the COVID-19 pandemic in China, those who had experiences in Wuhan were urged to quarantine for 14 days in designated facilities or homes. Therefore, those returning individuals might have regarded the quarantine as a discrimination signal. Furthermore, media-driven labeling of the unprecedented risk of COVID-19 in Wuhan, to some extent, had fueled discrimination toward these individuals, who had a similar experience with the residents in Hong Kong and Dallas during the SARS [43] and Ebola crises [44,45]. Consequently, they were prone to experience discrimination and stigmatization related to COVID-19, as they might have been blamed as potential “sources of infection”.

A significant association between the experience of discrimination and adverse psychological consequences were observed in this study. We found that the negative mental health outcomes were positively associated with COVID-19-related discrimination among returning college students in Wuhan. This finding is consistent with numerous previous studies revealing that individuals with perceived discrimination showed increased morbidity rates of negative mental health outcomes [46], especially a study conducted during the COVID-19 pandemic [40]. The experience of discrimination implied rejection, as well as the exclusion of targeted groups, and a threat of self-concept, thus spontaneously prompting detrimental psychological outcomes and lower quality of life [47].

Research had linked experiences of discrimination with detrimental psychological consequences, but less was known about the role of shame and internalized stigma as mediators. To this end, our study was sequentially targeted at exploring, prospectively, whether the linkage between discrimination and psychological wellbeing was accounted for by shame and internalized stigma. In this study, direct and indirect effects of the experience of discrimination, shame, and internalized stigma on negative mental health outcomes among participants were explored. Corroborating previous studies [40], the experience of discrimination was directly associated with high levels of negative mental health outcomes, and the effect sizes were substantial without considering the mediating effects of COVID-19-related shame and internalized stigma. However, further meditational path analyses surprisingly indicated that, except for an individual’s COVID-19-related distress, the experience of discrimination demonstrated a total indirect influence on mental health outcomes (anxiety, depression, insomnia), with shame and internalized stigma playing important mediating roles. In particular, several potential mechanisms, by which the experience of discrimination could affect the negative mental health outcomes, were observed. Shame and internalized stigma could separately mediate the impact of the experience of discrimination on negative mental health outcomes. This was consistent with previous studies [48,49] that internalized stigma could take a toll on mental health as a mediator. Meanwhile, our findings suggested that shame could directly influence the internalized stigma, thus, affecting negative mental health outcomes through their chain mediating path. This was in line with previous studies linking shame and internalized stigma to increased risks for negative mental health outcomes [20,50,51,52,53,54,55,56,57,58] in the context of HIV, obesity, schizophrenia, and marginalized groups. Multiple mediating analyses further revealed the underlying mechanisms and explained why discrimination could increase negative mental health outcomes. Previous studies confirmed the constructive role of coping with stigma on maintaining a positive self-concept [10] and self-esteem [59]. However, the discriminated individuals tended to experience higher shame and internalized stigma, which in turn implied the malfunction of coping strategies with stigma and discrimination. Consequently, negative mental health outcomes increased when low self-concept and self-esteem were shaped as a result of shame and internalized stigma. Taken together, we argued that a higher level of shame and internalized stigma appeared to occur successively without detecting when individuals were discriminated against in various ways, and became aware of the potential link, along with stereotypes with COVID-19, then directly affected the negative mental health outcomes.

The experience of discrimination could both directly and indirectly impact COVID-19-related distress, considering the mediator of shame and internalized stigma, since the COVID-19 pandemic had created an unprecedented panic [60], and the discrimination encountered by individuals could be profoundly impressed and enduringly related. However, it seemed to be unsuccessful in directly predicting a higher level of anxiety, depression, and insomnia since there were no significant direct effects observed, supporting the notion that shame and internalized stigma play an important role in determining the individual’s negative emotions [61]. To be more specific, being discriminated against—momently regarded as an unpleasant experience by individuals—did not necessarily prompt a higher level of anxiety, depression, and insomnia, unless shame and internalized stigma were perceived during the process in the long-term.

The study is not exempt from limitations. First, this study is exploratory, as the cross-sectional design prohibits inferring causal relationships among variables. Further research should utilize longitudinal data to explore causality. In addition, a single item was used to assess the experience of discrimination, and both internalized stigmatization and shame scales are short compared with other studies. Therefore, psychometric properties could not be fully established, although similar items and scales [40,41] had been used in previous studies. Future studies should use multi-item and context-specific instruments to access the experience of discrimination. Second, large-scale gatherings and dispensable mobilization were strictly prohibited due to the stern restrictions and sealed management implemented by universities in Wuhan. For this reason, we adopted the convenience sampling method, which may restrict the representativeness of our samples. Third, the findings of this study may not generally apply to other groups as the sample was non-randomly selected and limited to university students. Hence, further research can carry out among different groups suffering the stigma, especially in those areas with a weak medical system. Furthermore, follow-up qualitative studies, and a more sophisticated questionnaire design, are urgently needed to explore more specific effects of COVID-19-related discrimination in the future.

## 5. Conclusions

Our findings extended previous cross-sectional research and demonstrated that the experience of COVID-19-related discrimination was positively associated with shame and internalized stigma, which in turn predicted negative mental health outcomes over time. First, these findings suggested that there was a trade-off between the merits of mandatory measures and the risk of pervasive social tensions. Hence, policymakers were supposed to balance tensions between discrimination as well as stigma mitigation and the COVID-19 containment so as to eliminate discrimination. Second, misinformation and negative stereotypes are key drivers of fear and stigma. The implication of this mediation analysis is that inclusive language and less stigmatizing terminology are urgently needed to prevent stigmatization, and an emerging disease should not be attached to location or ethnicity. Meanwhile, it would be important for social influencers and community leaders to create public awareness of fighting the pandemic, without stigma, through social media or the online community, and construct a supportive and tolerant environment to help subdue unverified rumors and alleviate stigmatization among individuals who exhibit a potential and perceived linkage with COVID-19. Third, college students who have been affected by COVID-19 should not be reckoned with. Tailored intervention strategies, such as large-scale COVID-19-related psychoeducation programs and accessible psychological counseling, were needed to mitigate individual negative mental health outcomes on an intrapersonal level in college settings.

## Figures and Tables

**Table 1:** Demographic characteristics and discrimination relative information.

Variable	Categories	n	%
Sex	Male	463	46.53
	Female	532	53.47
Age	18–21	421	42.31
	22–25	501	50.35
	26–29	63	6.33
	30 and above	10	1.01
Years expected to graduate	Within 1 year	339	34.07
	Within 2 years	328	32.96
	Within 3 years	240	24.12
	4 years and above	88	8.84
Have you ever been discriminated against because of your experience in Wuhan?	Yes	407	40.90
	No	588	59.10
Source of discrimination	Community	312	76.66
	Friends and relatives around	221	54.30
	Social Media	134	32.92
	Restaurants, hotels, hospitals, and other facilities	90	22.11
	Administrative Departments and law enforcement agencies	90	22.11
	Railway transportation and airport department	55	13.51
Form of discrimination	Social avoidance	277	68.06
	Personal information leakage	220	54.05
	Abusive expressions	187	45.95
	Deliberately complicated examination procedure	157	38.57
	Others	19	4.67
	Body conflicts	17	4.18

**Table 2:** Means, standard deviations, and bivariate correlations among variables.

Variable	M	SD	1	2	3	4	5	6	7
1. Discrimination			1						
2. Shame	8.24	4.74	0.116 ***	1					
3. Internalized stigma	36.37	12.45	0.390 ***	0.406 ***	1				
4. Anxiety	11.38	4.30	0.084 **	0.300 **	0.270 ***	1			
5. Distress	15.84	4.98	0.175 ***	0.316 ***	0.351 ***	0.443 ***	1		
6. Depression	14.21	5.18	0.099 **	0.334 ***	0.279 ***	0.716 ***	0.454 ***	1	
7. Insomnia	13.80	5.50	0.101 **	0.282 ***	0.265 ***	0.518 ***	0.441 ***	0.641 ***	1

Note: n = 995, ** *p* < 0.01, *** *p* < 0.001.

**Table 3:** Regression results.

Regression Model		Fitting Index			Significance of Coefficients	
Outcome Variable	Independent Variable	R	R^2^	F	β	t
Shame	Sex	0.1502	0.0226	5.712	−1.0219	−3.4045
	Age				0.5129	2.1465 *
	YEG				−0.083	−0.5266
	Discrimination				0.7683	2.5197 *
Internalized Stigma	Sex	0.5934	0.3521	107.515	0.4303	0.6666
	Age				2.0311	3.9665 ***
	YEG				−0.0816	−0.2421
	Discrimination				7.811	11.9435 ***
	Shame				1.1975	17.6241 ***
Anxiety	Sex	0.3969	0.1575	30.795	0.359	1.4108
	Age				−0.2052	−1.0089
	YEG				−0.2428	−1.8277
	Discrimination				0.0391	0.1418
	Shame				0.2636	8.5866 ***
	Internalized sigma				0.0552	4.4006 ***
Distress	Sex	0.4207	0.177	35.408	0.1259	0.4321
	Age				0.0476	0.2045
	YEG				−0.4952	−3.2545 **
	Discrimination				0.7698	2.4375 *
	Shame				0.2536	7.2125 ***
	Internalized stigma				0.0787	5.4841 ***
Depression	Sex	0.4487	0.2014	41.518	0.8699	2.9115 **
	Age				0.1959	0.82
	YEG				−0.3077	−1.973 *
	Discrimination				0.0594	0.1836
	Shame				0.3848	10.6758 ***
	Internalized stigma				0.0569	3.8697 ***
Insomnia	Sex	0.4036	0.1629	32.042	0.561	1.7289
	Age				1.0709	4.1285 ***
	YEG				−0.0996	−0.588
	Discrimination				0.1528	0.4347
	Shame				0.3038	7.7609 ***
	Internalized stigma				0.067	4.1921 ***

Note: n = 995, * *p* < 0.05, ** *p* < 0.01, *** *p* < 0.001. YEG, years expected to graduate.

**Table 4:** Direct and indirect effects of coronavirus disease 2019 (COVID-19)-related discrimination on anxiety, distress, depression, and insomnia through the mediating effects of shame and internalized stigma.

Model	Path	Effect	Boot SE	Boot 95%CI	Effect Size
D on anxiety	Direct effect	0.0391	0.2757	−0.5020, 0.5802	5.41%
	Indirect effect				
	Total	0.6841	0.1529	0.3889, 0.9918	94.59%
	D→S→Anxiety	0.2025	0.0887	0.0361, 0.3899	28.00%
	D→IS→Anxiety	0.4308	0.1127	0.2204, 0.6633	59.57%
	D→S→IS→Anxiety	0.0507	0.0254	0.008, 0.1077	7.01%
D on distress	Direct effect	0.7689	0.3158	0.1501, 1.3896	46.54%
	Indirect effect				
	Total	0.8822	0.1795	0.5511, 1.2642	53.40%
	D→S→Distress	0.1949	0.0822	0.0411, 0.3669	11.80%
	D→IS→Distress	0.6149	0.132	0.3761, 0.8908	37.22%
	D→S→IS→Distress	0.0724	0.0366	0.0141, 0.1597	4.38%
D on depression	Direct effect	0.0594	0.3238	−0.5759, 0.6948	6.97%
	Indirect effect				
	Total	0.7929	0.1904	0.4261, 1.1704	93.03%
	D→S→Depression	0.2957	0.1266	0.0516, 0.5541	34.69%
	D→IS→Depression	0.4448	0.126	0.2046, 0.7034	52.19%
	D→S→IS→Depression	0.0524	0.0272	0.0077, 0.1135	6.15%
D on insomnia	Direct effect	0.1528	0.3516	−0.5372, 0.8428	15.73%
	Indirect effect				
	Total	0.8184	0.1954	0.458, 1.2168	84.27%
	D→S→Insomnia	0.2334	0.1025	0.0482, 0.4457	24.03%
	D→IS→Insomnia	0.5233	0.1435	0.2567, 0.8183	53.88%
	D→S→IS→Insomnia	0.0616	0.0325	0.0107, 0.1367	6.34%

Note: D, the experience of discrimination; S, shame; IS, internalized stigma. Models were adjusted for sex, age, and years expected to graduate.
